# P-selectin glycoprotein ligand-1 and cardiovascular diseases: from a general perspective to an HIV infection context

**DOI:** 10.3389/fcvm.2025.1521158

**Published:** 2025-02-18

**Authors:** Silvere D. Zaongo, Yuxia Song, Yaokai Chen

**Affiliations:** ^1^ Department of Infectious Diseases, Chongqing Public Health Medical Center, Chongqing, China; ^2^ Department of Infectious Diseases, The Sixth People’s Hospital of Xinjiang Uygur Autonomous Region, Urumqi, China

**Keywords:** P-selectin glycoprotein ligand-1, HIV, cardiovascular diseases, people living with HIV, inflammation

## Abstract

Globally, cardiovascular diseases (CVDs) are a leading cause of death as they are responsible for the loss of at least 17 million lives annually. It has been established that the pathogenesis of CVDs is strongly associated both with inflammation as well as with inflammatory markers (proteins, cytokines, amongst others). In this perspective, the role of one of these proinflammatory proteins, referred to as P-selectin glycoprotein ligand (PSGL)-1, is of particular interest. Indeed, contemporary evidence points to the fact that P-selectin glycoprotein ligand (PSGL)-1 plays a critical role in the development of CVDs via its interactions with P-selectin, L-selectin, and/or E-selectin. However, due to the dearth of published contemporary research concerning PSGL-1 expression in people living with HIV (PLWH), it remains challenging to comprehensively investigate this area of study, although potential clues exist in the literature which may serve as potential directions for future investigations. Hence, in the first part of this article, a scoping review of the literature regarding the role of PSGL-1 in the development of CVDs is provided. Then, in the second part, observations concerning PSGL-1 expression in PLWH receiving ART are presented and interpreted. Through this work, we hope that increased attention will be directed towards the screening of PSGL-1 expression, which we believe may serve as a reliable biomarker to predict the presence and evolution of CVDs in PLWH.

## 1 Introduction

Cardiovascular diseases (CVDs) are a group of disorders that affect the heart and the blood vessels. In 2019, the world health organization (WHO) estimated that 17.9 million people died from CVDs worldwide, which represented 37% of all global deaths. Moreover, it is known that CVDs may result from an unhealthy diet, physical inactivity, tobacco use, and injudicious use of alcohol (1). In contemporary times, cumulative evidence points to the critical role of prevailing and sustained inflammation in the pathogenesis of CVDs (2–6). Indeed, immune cells are considered as fundamental players in the development of CVDs, and more importantly, they are considered as critical elements which have the potential to be targeted in the quest to treat CVDs. As such, one of the fundamental investigative publications in this realm of research endeavor was presented by Ridker et al. (7), who demonstrated that antagonization of the activities of interleukin (IL)-1β [using Canakinumab (a specific monoclonal antibody)] may effectively (i) lower rates of recurrent CVD in patients with previous myocardial infarction, (ii) lower existing high levels of C-reactive protein in these patients, and (iii) decrease circulating levels of IL-6. From the preceding general commentary concerning the role of inflammation and innate immunity in the development of CVDs, we believe that the role of one specific proinflammatory protein, known as P-selectin glycoprotein ligand (PSGL)-1, should be considered with much more diligence.

PSGL-1 is a transmembrane proinflammatory receptor expressed on plasma cells such as monocytes/macrophages, neutrophils, T-cells, and B-cells, amongst others. It is essential (i) for the recruitment of immune cells to inflamed tissues and (ii) in the immunological response to infectious diseases, such as HIV infection. Over the years, researchers have revealed a positive correlation between genetic variations in the PSGL-1 gene (SELPG) and the ligand P-selectin gene (SELP), and CVDs (8–11). The knowledge that PSGL-1 expression is related to inflammation as well as the fact that inflammation results in CVDs makes it fundamental, therefore, to clearly understand whether an infection leading to sustained chronic inflammation, such as HIV infection, has the capacity to modulate PSGL-1 expression and potentially orchestrate the onset and development of CVDs in people living with HIV (PLWH). In other words, is PSGL-1 involved in the development of CVDs in PLWH? Thus far, to the best of our knowledge, such investigative endeavor does not exist in the contemporary literature. Nonetheless, it is now well understood that HIV infection increases the risk of development of CVDs despite the widespread availability of modern antiretroviral therapy (ART). Hence, the proposition concerning the influence of PSGL-1 in the context of CVD may well represent an interesting and justifiable target to potentially mitigate the onset and development of CVDs in PLWH.

Herein, we hypothesize that PSGL-1 expression may serve as a reliable biomarker to predict the presence and evolution of cardiovascular diseases in PLWH, especially those on ART. To further investigate this hypothesis, we have presented this article in two parts. The first part reviews existing knowledge that has been reported in the contemporary literature with respect to the implications of PSGL-1 in the pathogenesis of CVDs. This serves to identify whether PSGL-1 levels are indeed associated with CVDs or not. Then, in the second part, our hypothesis concerning the potential implications of PSGL-1 levels on the onset of CVDs in ART-treated HIV-positive individuals is proposed. The trend of PSGL-1 expression observed in HIV-infected individuals by other research teams as well as our group is presented in the second part, and these results are juxtaposed against the overall knowledge gleaned from the contemporary literature. These observations are then interpreted and elaborated upon.

## 2 PSGL-1 is involved in the pathogenesis of cardiovascular diseases

Several past publications indicate that the presence and overexpression (excessive expression) of PSGL-1 contributes to the development of CVDs. These publications inform that hypertension, atherosclerosis, thrombus, stroke, and chronic heart disease are intriguingly associated with PSGL-1 expression.

### 2.1 Hypertension

In their investigations, Yang et al. (12), have observed that PSGL-1 may be involved in the development of salt-sensitive hypertension. Indeed, in comparing mice without and with PSGL-1 (PSGL-1^−/−^ and PSGL-1^+/+^, respectively) and fed with a high salt diet, they observed that PSGL-1^+/+^ mice developed high blood pressure. Furthermore, PSGL-1^+/+^ mice were observed to have an overexpression of vascular injury markers [monocyte chemotactic protein (MCP)-1, endothelin (ET)-1, and Von Wille-brand factor (VWF)] in addition to increased serum levels of inflammatory markers [IL-6, IL-1β, TNF-α, mainly secreted by activated macrophages and T-cells (13)]. Specifically, Yang et al., opine that PSGL-1 may participate in regulation of vascular injury via inflammation resulting from a high salt intake. As such, they suggest that the main target that should be pursued in the quest to treat and control hypertension should be vascular injury. In this regards, PSGL-1 expression, which is known for its roles in (i) leukocyte activation, (ii) promotion of rolling and extravasation, and (iii) contribution to vascular injury (14) may contribute significantly to and/or aggravate endothelial injury, as observed by Daniel et al. (15). Consequently, PSGL-1 expression may be strongly associated with the pathophysiology of salt-sensitive hypertension. On the one hand, this is supported by the evidence of Yang et al., showing that in the absence of PSGL-1 (and in the context of a high salt environment) no high blood pressure is observed. On the other hand, and still in the context of a high salt environment, the adhesion of peripheral blood mononuclear cells (PBMCs, including leukocytes) to endothelial cells (via PSGL-1/E-selectin) and platelets (via PSGL-1/P-selectin engagement) is significantly prevented in PSGL-1 knockout mice. Similarly, Ajayi and Obayuwana (16) observed that a high salt diet (in addition to raising arterial blood pressure) leads to an overexpression of both, PSGL-1 and intracellular adhesion molecule (ICAM)-1, on leukocytes sampled from Sprague-Dawley rats. In mice, Wang et al. (17), have also demonstrated that the deficiency of PSGL-1 on plasma cells may attenuate angiotensin-II-induced hypertension, and their findings suggest that this is potentially mediated by the reduced levels of IL-17 observed in PSGL-1^−/−^ mice. Furthermore, Parissis et al. (18), have observed that compared to healthy controls, patients with hypertension have higher expression of P-selectin, which is the primary ligand of PSGL-1 (19). However, hypertensive patients with normal cholesterol levels appear to have lower expression of P-selectin than hypertensive patients who are hypercholesterolemic (18). This suggests that PSGL-1 expression may be associated with cholesterol levels in addition to its involvement in the pathogenesis of hypertension. Intriguingly, it seems that the overexpression of PSGL-1 on plasma cells is not always a risk factor for hypertension. Indeed, PSGL-1 expression, when reduced, has been observed by Gonzalez-Tajuelo et al., in their study using female mice, to be associated with the pathogenesis of pulmonary arterial hypertension (20).

### 2.2 Atherosclerosis

It has been demonstrated that PSGL-1 and P-selectin attachment contributes to vascular injury and results in exacerbation of atherosclerosis (14). In their study, Ye et al. (21), have demonstrated that in mice, deficiency of PSGL-1 or P-selectin may attenuate the development of atherosclerosis. The observations of Ye et al., align well with those of previous research groups who have made similar observations (14, 22–24). Furthermore, it is known that dendritic cells are present in high numbers in an atherosclerotic vessel wall (25, 26). Ye et al. (21), investigated the behavior of these dendritic cells with and without PSGL-1 or P-selectin. Interestingly, Ye et al. (21), have observed that in the absence of PSGL-1 or P-selectin, the maturation of dendritic cells is inhibited in the contexts of inflammation and hypercholesterolemia. Of particular note is the fact that they also observed that in the absence of PSGL-1 or P-selectin, the homing of dendritic cells to atherosclerotic lesions is significantly reduced, even in the context of hypercholesterolemia and inflammation. Physiologically, the binding of P-selectin on platelets to PSGL-1 present on dendritic cells initiates dendritic cell activation via the toll like receptor (TLR)-4 pathway. Subsequently, the dendritic cell initiates behavior [associated with the myeloid differentiation primary response 88 (MyD88)-dependent and the MyD88-independent TLR4 pathways] such as (i) phenotypic maturation, (ii) enhanced secretion of proinflammatory cytokines, (iii) communication with T-cells, and (iv) adhesion and migration, which contributes to the collective development and progression of atherosclerosis (21). Other researchers have noted that the absence of PSGL-1 in the low density lipoprotein receptor murine model (used to study hypocholesterolemia) significantly contributes to inhibition of the progression of atherosclerosis on the one hand, and also has been observed to regulate the metabolism of elements such as glucose, lipids, amino acids, and phospholipids (22) on the other hand.

### 2.3 Thrombus

The role of PSGL-1 in thrombus formation has now been recognized, mainly as an outcome of animal experimentation (27–30). In animal models expressing PSGL-1, thrombus formation is initiated via binding of PSGL-1 present on immune cells and P-selectin present on activated platelets (31, 32). Indeed, the PSGL-1-P-selectin axis mediates the formation of cell aggregates (33). Mechanically, the attachment of PSGL-1 to P-selectin favors the release of procoagulant microparticles and tissue factor, which is a protein molecule that plays a key role in the blood clotting and thrombosis cascade. In turn, tissue factor activates the coagulation cascade and mediates inflammatory responses. Then, once PSGL-1 on leukocytes binds to P-selectin on activated platelets in thrombi, the aggregated leucocyte/platelet complex further favors the recruitment of procoagulant microparticles to the thrombus being formed. As these interactions progress and evolve, there is a progressive accumulation of thrombin and fibrin deposition, and ultimately a stabilization of the formed thrombus (34–39). Other than utilization of P-selectin, PSGL-1 may also mediate cell aggregation via L-selectin expressed on leukocytes, which also induces thrombus formation (40). In humans, Ozaki et al., have observed that PSGL-1 is overexpressed on monocytes (CD14++CD16+) from patients with plaque rupture or an intracoronary thrombus (41). It is thus a justifiable exercise to hypothesize that a lack or deficiency of PSGL-1 would be protective against the development of thrombosis. Interestingly, it has been observed in past studies that the absence of PSGL-1 is protective against arterial and venous thrombosis (42–44). In their investigation, Wang et al. (45), have observed that the administration of anti-PSGL-1 antibodies may also mitigate endotoxemia-induced thrombotic events in a murine model. Indeed, PSGL-1, by promoting attachment of platelets and leukocytes, favors their infiltration, the activation of endothelial cells, the expression of tissue factor on leukocytes, and the deposition of fibrinogen, which results in inflammation and coagulopathy. Thus, the utilization of anti-PSGL-1 antibodies may represent a potentially important strategy to mitigate the onset of thrombus formation.

### 2.4 Heart related diseases

Ozaki et al., have observed a significant overexpression of PSGL-1 on monocytes (CD14+/CD16+) sampled from patients with acute myocardial infarction (41). In addition to myocardial infarction, PSGL-1 is also known to be involved in the manifestation of coronary heart disease, which is defined as a cascade of coronary atherosclerotic events in which lipid and fibrous matrix become deposited on the walls of coronary arteries to form atheromatous plaques (46–48). As an example, Hansson et al. (49), have demonstrated that inflammation triggers the recruitment/adhesion of leukocytes by cellular adhesion molecules (P-, E-, and L-selectin) via PSGL-1, which is a critical step in the development of coronary heart disease. Similarly, Kitamura et al. (50), have observed that compared to healthy controls, patients with acute coronary syndrome an overexpression of PSGL-1 on their CD4+ T-cells. In such patients, activated PSGL-1 on CD4+ T-cells (when engaged by P-selectin or E-selectin) may adhere to endothelial cells and induce their apoptosis via activated caspase-3 (50). Interestingly, the preceding scenario is mitigated by administration of an antibody specific to PSGL-1, allowing conclusions to be made around the negative contribution of PSGL-1 to the pathogenesis of acute coronary syndrome. From the preceding evidence, it is safe to state that the formation of platelet and leukocyte complexes (via PSGL-1 and P-selectin bonding) are implicated in several CVDs (51). Hence, Fernandes et al. (52), have observed that the blockade of PSGL-1 alone is sufficient to significantly mitigate the formation of platelet-monocyte complexes (PMCs) in patients with coronary syndromes (patients with acute coronary syndrome and those with complications after percutaneous coronary intervention). More specifically, the utilization of anti-PSGL-1 (KPL-1) reduces PMC formation by 90% (52). This suggests that the formation of PMCs, in general, is dependent on PSGL-1, and neutralization of PSGL-1 may be beneficial in the treatment of coronary syndromes.

In a recent study, Wu et al. (53), have observed that the development of aortic aneurysm is positively associated with PSGL-1 levels in humans and mice. Indeed, leukocytes sampled from patients and mice with aortic aneurysm have been observed to have overexpression of PSGL-1. In PSGL-1^−/−^ mice, the preceding research team observed that deficiency of PSGL-1 contributes significantly to reduce both the incidence and the severity of aortic aneurysm. This may be explained by the absence of PSGL-1, which results in reduced adhesion between PSGL-1 and the other respective adhesion molecules. Subsequently, there is a reduced quantum of leukocyte-platelet or leukocyte-endothelial complexes (which Wu et al., observed initially pass through the NF-kB pathway). The aftermath of this manifests as reduced inflammatory cell infiltration and decreased inflammatory factor expression. Thus, Wu et al. (53), concluded that PSGL-1 plays a major role in inflammatory cell migration and recruitment, and as such, PSGL-1 blockade or inhibition may be an important option to explore in the quest to prevent or treat aortic aneurysm.

### 2.5 Cerebrovascular disease and stroke

With the knowledge that PSGL-1 mediates and regulates the recruitment and activation of multiple types of leukocytes through interactions with selectins (54), Wang et al. (44), have investigated the effect of PSGL-1 inhibition on the occurrence of stroke in a murine model of lupus. They observed that the absence of PSGL-1 is protective against stroke, as the expected infiltration of neutrophils and macrophages at cerebral infarction sites were observed to be significantly reduced. Thus, the increased stroke size usually associated with lupus is prevented in PSGL-1^−/−^ mice. This outcome serves to open the gate to speculation concerning potential strategies which may target PSGL-1 in the management of patients at high risk of stroke in particular, and of acute vascular complications in general. In a recent publication, Li et al. (55), have extensively explored the implications of PSGL-1 expression in the pathogenesis of ischemic stroke. Briefly, they have observed that neutrophil activation is initiated by PSGL-1 binding to P-selectin. Thus, the activated neutrophil initiates the upregulation of peptidyl arginine deiminase-4 (PAD4) and promotes the formation of citrullinated histone (56, 57). Consequently, this tandem contributes to the release of neutrophil extracellular traps (NETs) (58, 59), even in the absence of infection. Interestingly, NETs are recognized for their role in neuronal death through exacerbation of inflammation (thromboinflammation) and neurological damage in ischemic brain tissue. Notably, some researchers (60–62) have observed that the inhibition of NET formation through suppression/inhibition of PSGL-1 on neutrophils may prevent thrombosis and the infiltration of immune cells. Thus, if PSGL-1 is one of the root causes of NET formation, the inhibition of its expression on neutrophils may be considered as a viable strategy to prevent thrombosis, immune cell infiltration, inflammation, and potentially therefore CVDs in general.

Table 1 summarizes the role of PSGL-1 in the development of CVDs.

**Table 1:** Studies reporting the implications of PSGL-1 in the pathophysiology of cardiovascular diseases.

Type of CVD	Description of PSGL-1 implication	Type of association	Study subjects	References
Hypertension	The absence of PSGL-1 prevents the onset of hypertension and vascular injury via inflammation resulting from a high salt intake	Positive	Mice	(12)
	The deficiency of PSGL-1 on plasma cells may attenuate angiotensin-II-induced hypertension	Positive	Mice	(17)
	Patients with hypertension display overexpression of P-selectin, which is the primary ligand of PSGL-1	Positive	Humans	(18)
	Reduced expression of PSGL-1 is associated with the pathogenesis of pulmonary arterial hypertension	Negative	Mice	(20)
Atherosclerosis	PSGL-1 (on monocytes) may exacerbate the development of atherosclerosis when engaged in complex formation with platelets via P-selectin	Positive	Mice	(14)
	The absence of PSGL-1 or P-selectin inhibits the maturation of dendritic cells. Thus, without PSGL-1 or P-selectin, the homing of dendritic cells to atherosclerotic lesions is significantly reduced. Therefore, PSGL-1 prevents the development of atherosclerosis in the context of hypercholesterolemia and inflammation.	Positive	Mice	(21)
	The absence of PSGL-1 significantly inhibits the progression of atherosclerosis	Positive	Mice	(22)
Thrombus	The binding of PSGL-1 present on leucocytes and P-selectin present on activated platelets leads to formation of leucocyte-platelet aggregates, which initiate the formation of thrombus.	Positive	Mice	(31, 32)
	The presence of PSGL-1 may also mediate cell aggregation via L-selectin expressed on leukocytes, which also induces thrombus formation	Positive	Human, mouse, and rat	(40)
	PSGL-1 is overexpressed on monocytes from patients with plaque rupture or an intracoronary thrombus	Positive	Humans	(41)
	Thrombus formation may be mitigated through administration of anti-PSGL-1 antibodies, which neutralize the formation of leukocyte-platelet or leukocyte-leukocyte aggregates.	Positive	Mice	(45)
Heart-related diseases	Patients with acute myocardial infarction have an overexpression of PSGL-1 on their monocytes	Positive	Humans	(41)
	The recruitment/adhesion of leukocytes by cellular adhesion molecules (P-, E-, and L-selectin) via PSGL-1, is a critical step in the development of coronary heart disease	Positive	Humans	(50)
	The blockade of PSGL-1 alone may significantly mitigate the formation of platelet-monocyte complexes in patients with coronary syndromes	Positive	Humans	(52)
	In mice, the deficiency of PSGL-1 contributes significantly to reduce both the incidence and the severity of aortic aneurysm, while leukocytes from humans and mice with aortic aneurysm have overexpression of PSGL-1	Positive	Humans and mice	(53)
Stroke	The infiltration of neutrophils and macrophages at cerebral infarction sites is significantly reduced in the absence of PSGL-1. Thus, the absence of PSGL-1 is protective against stroke.	Positive	Mice	(44)
	Neutrophil activation and the release of NETs are initiated by PSGL-1 binding to P-selectin. Unfortunately, NETs are recognized for their contribution in neuronal death and neurological damage.	Positive	Mice	(55, 60–62)

**Figure 1:**
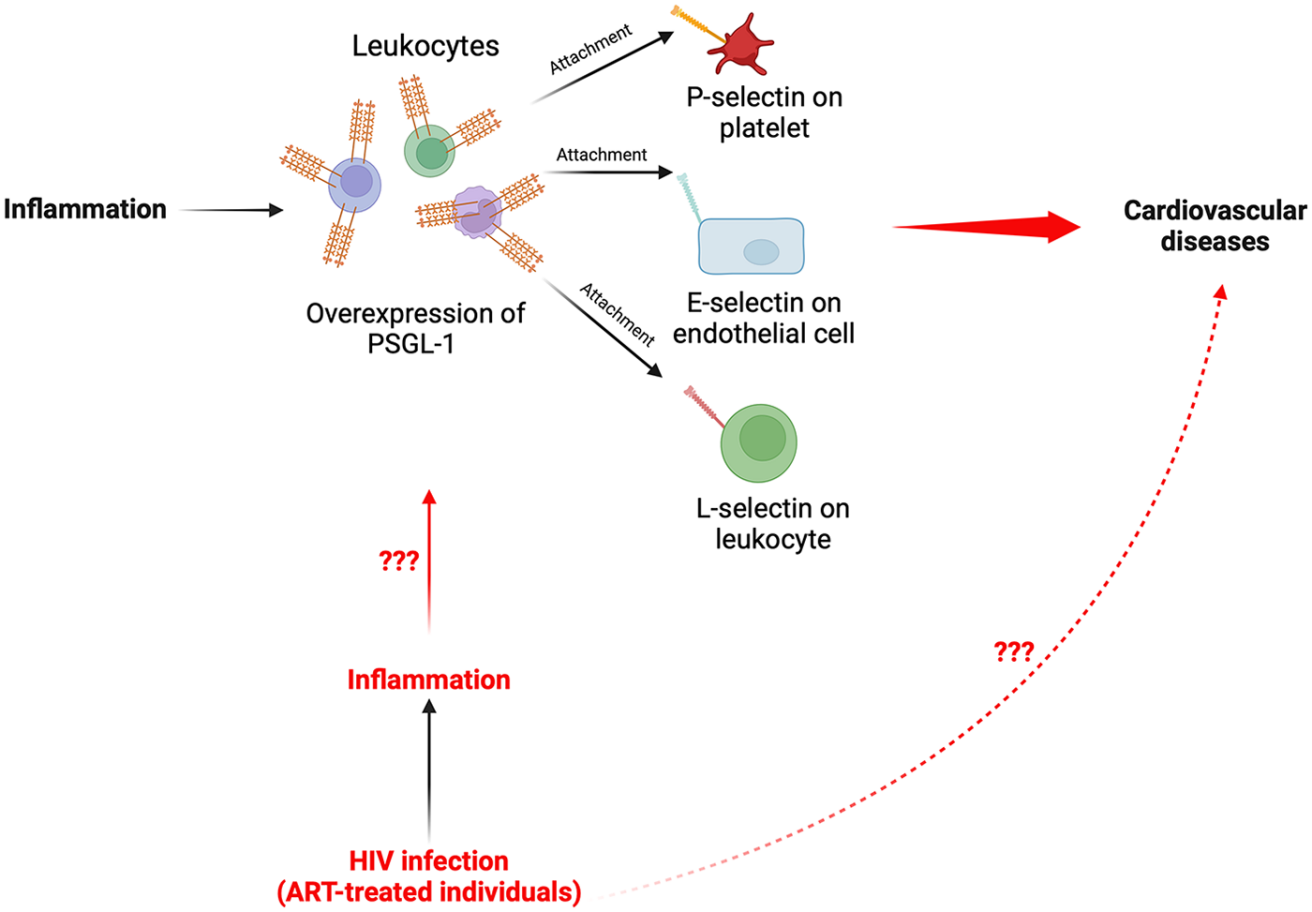
Schematic representation summarizing the proposed pathway of PSGL-1 from inflammation to CVDs in PLWH. Elucidation of the fundamental mechanisms whereby the chronic inflammation, which persists in ART-treated individuals, may induce an overexpression of PSGL-1 and potentially contribute to development of CVDs in PLWH remain to be investigated.

In light of the preceding contemporary research information, it is likely that an overexpression of PSGL-1 on immune cells may well induce the development of CVDs. With the knowledge that PSGL-1 is a proinflammatory receptor, it is therefore essential to understand PSGL-1 expression in the context of sustained chronic inflammation (as is ubiquitous in HIV infection), and particularly in ART-treated HIV-infected individuals. Contemporary evidence indicates that in ART-treated people living with HIV (PLWH), immunological nonresponders (INRs) are more likely to be affected by non-AIDS comorbidities (63, 64) and/or develop metabolic diseases, including CVDs (65–67). Some have postulated that the enduring chronic inflammation, the inappropriate immune responses (68–71), and the gut microbial translocation and dysbiosis (72) in PLWH may contribute to these outcomes. Cognizant of the implications of PSGL-1 in the pathogenesis of CVDs, it is thus compelling to be curious with respect to the expression of PSGL-1 in ART-treated PLWH. This curiosity may possibly stimulate future investigations which explore the emergence and development of CVDs in the context of HIV infection. The hypothesis that PSGL-1 overexpression due to inflammation may lead to CVDs in ART-treated individuals is visually represented in Figure 1.

## 3 PSGL-1 expression in ART-treated HIV-infected individuals may serve as a potential marker for CVDs

### 3.1 Chronic inflammation may influence PSGL-1 overexpression in ART-treated individuals

**Figure 2:**
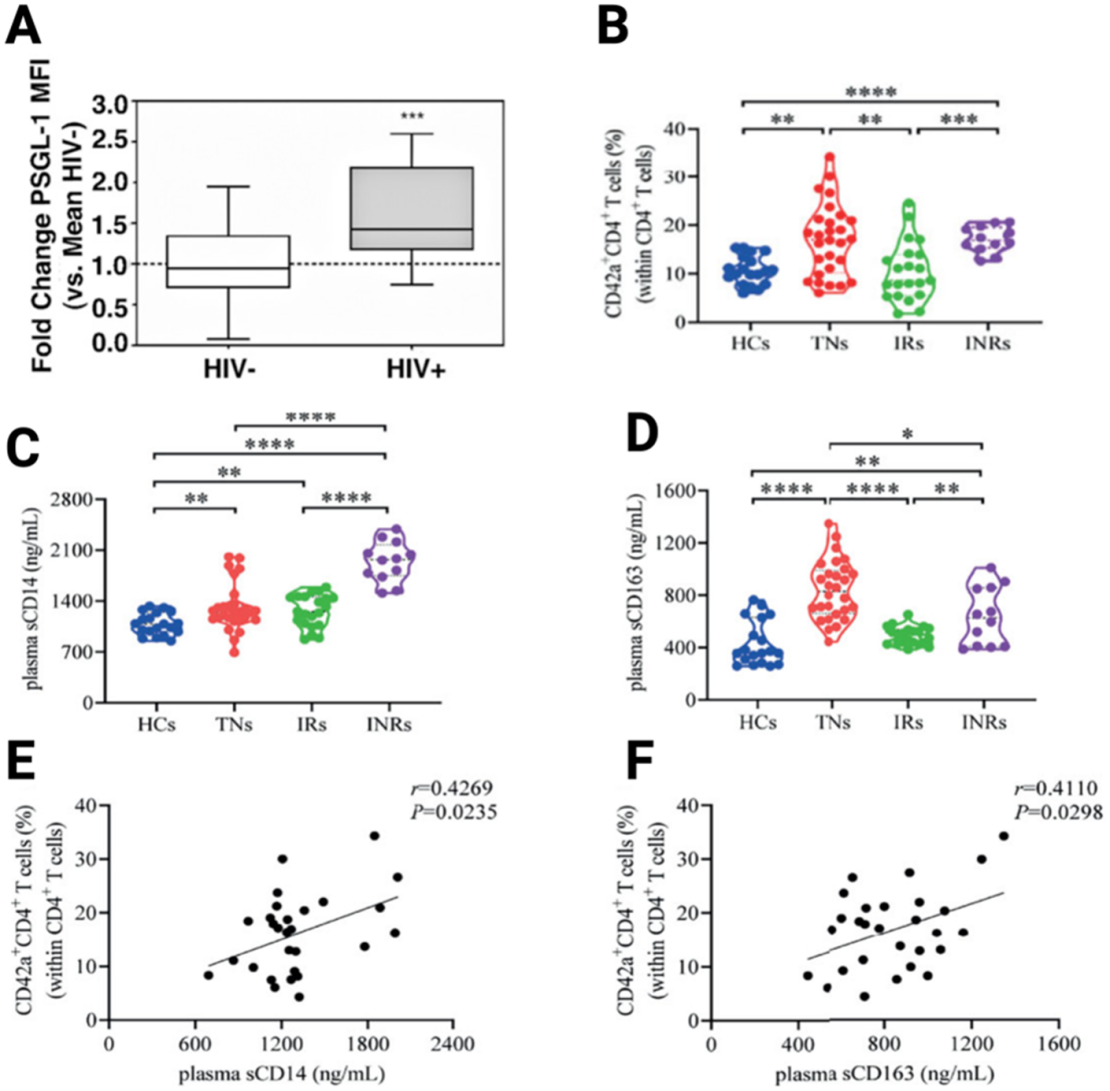
Overexpression of PSGL-1 in ART-treated HIV-positive individuals **(A)**, its implications in the formation of platelet-CD4+ T-cell aggregates **(B)**, and the existing correlations between inflammation marker levels **(C,D)** and cell aggregates **(E,F)**. HIV-: HIV negative; HIV+: HIV positive; HCs: healthy controls; TNs: treatment naïve; IRs: immunological responders; INRs: Immunological nonresponders. From Connor et al. (82) and Dai et al. (76).

In this era of modern ART, although HIV replication is effectively repressed in ART-treated PLWH, background inflammation remains persistent and enduring in ART-treated individuals. Some believe that HIV-associated gut dysbiosis syndrome and the consequent leaky gut, which subsequently induces the release of microbial products into the bloodstream, represents one of the leading causes of the persisting chronic inflammation (73–75). The chronic inflammation is observed both in people who achieve immune reconstitution (or immunological responders: IRs) as well as in those who display incomplete immune reconstitution (immunological nonresponders: INRs). Notably, INRs display higher levels of inflammation compared to IRs. For example, Dai et al. (76), have observed higher levels of plasma markers of inflammation [such as soluble CD14 (sCD14) and sCD163] in the plasma of INRs compared to IRs (*p* < 0.05; Figure 1). Similarly, Dunham et al. (77), have shown that compared to IRs, the markers of systemic inflammation (TNF and IL-6), observed in INRs are significantly elevated. The persistent chronic inflammation in ART-treated individuals is of further concern as past evidence has demonstrated that PSGL-1 expression positively correlates with inflammation. Indeed, it has been established that inflammation provokes higher expression of PSGL-1 on immune cells (78–81), which in turn is utilized to translocate immune cells to potential sites of inflammation. Thus, one may speculate that the chronic inflammation in ART-treated individuals, as is apparent by elevated levels of plasma markers of inflammation in blood, may well induce overexpression of PSGL-1 on plasma cells. To illustrate this point, Connor et al. (82), have observed higher expression of PSGL-1 on monocytes from ART-treated HIV positive individuals with undetectable viral loads (Figure 2A). Conversely, another team (82) has reported that PSGL-1 levels are decreased in PLWH. However, the preceding team reported observations made only in ART-naïve patients. Thus, it is likely, upon further investigations, that the overexpression of PSGL-1 in ART-treated individuals may not only be limited to monocytes, but may extend to other plasma cells such as CD4+ cells, CD8+ cells, and neutrophils, amongst others.

Today, it is known that PSGL-1 expression may be induced by interferon gamma (IFN-γ) (83), IL-12 (84), sCD40 (82), and glutamate (82). Perhaps other proinflammatory cytokines, proteins, or microbial translocated products may modulate PSGL-1 expression as well; however, only future studies will help to further expand the preceding list. Cognizant of the implications of PSGL-1 expression for the onset of CVDs, it is therefore necessary to examine the implications of PSGL-1 overexpression in ART-treated individuals, and the potential association of PSGL-1 overexpression with CVDs.

### 3.2 PSGL-1, cell aggregates, and CVDs in ART-treated individuals

Cognizant of the overexpression of PSGL-1 in plasma cells of ART-treated individuals, it is critical to understand the implications of this in terms of physiology. As such, one Chinese research group has provided insightful clues. Indeed, in the ART-treated context, Dai et al. (76), have observed that there are significantly higher levels of platelet-CD4+ T-cell aggregates in INRs than in IRs (Figure 2B). Additionally, Dai et al., observed that INRs express higher levels of sCD163 and sCD14 (Figures 2C,D), levels of which positively correlate with platelet-CD4+ T-cell aggregates (Figures 1E,F). Notably, platelet-CD4+ T-cell aggregates are formed through the attachment of P-selectin on platelets and PSGL-1 on CD4+ T-cells. This is only a partial consideration as it is also known that PSGL-1 may also attach to L-selectin on leukocytes and E-selectin on endothelial cells (85). From this picture provided by Dai et al. (76), it is likely that in the HIV infection context, the overexpression of PSGL-1 in ART-treated individuals may provoke increased formation of platelet-CD4+ T-cell aggregates which are unfavorable to CD4+ T-cell survival, and may potentially induce the development of CVDs as well, when other varieties of plasma cells are taken into consideration. Indeed, the markers considered by Dai et al., (sCD163 and sCD14) as well as the sCD40 marker, are also known to be associated with CVDs (4–6). The preceding evidence sparked our curiosity and thus motivated further investigations by our group.

#### 3.2.1 Context of the preliminary study

**Figure 3:**
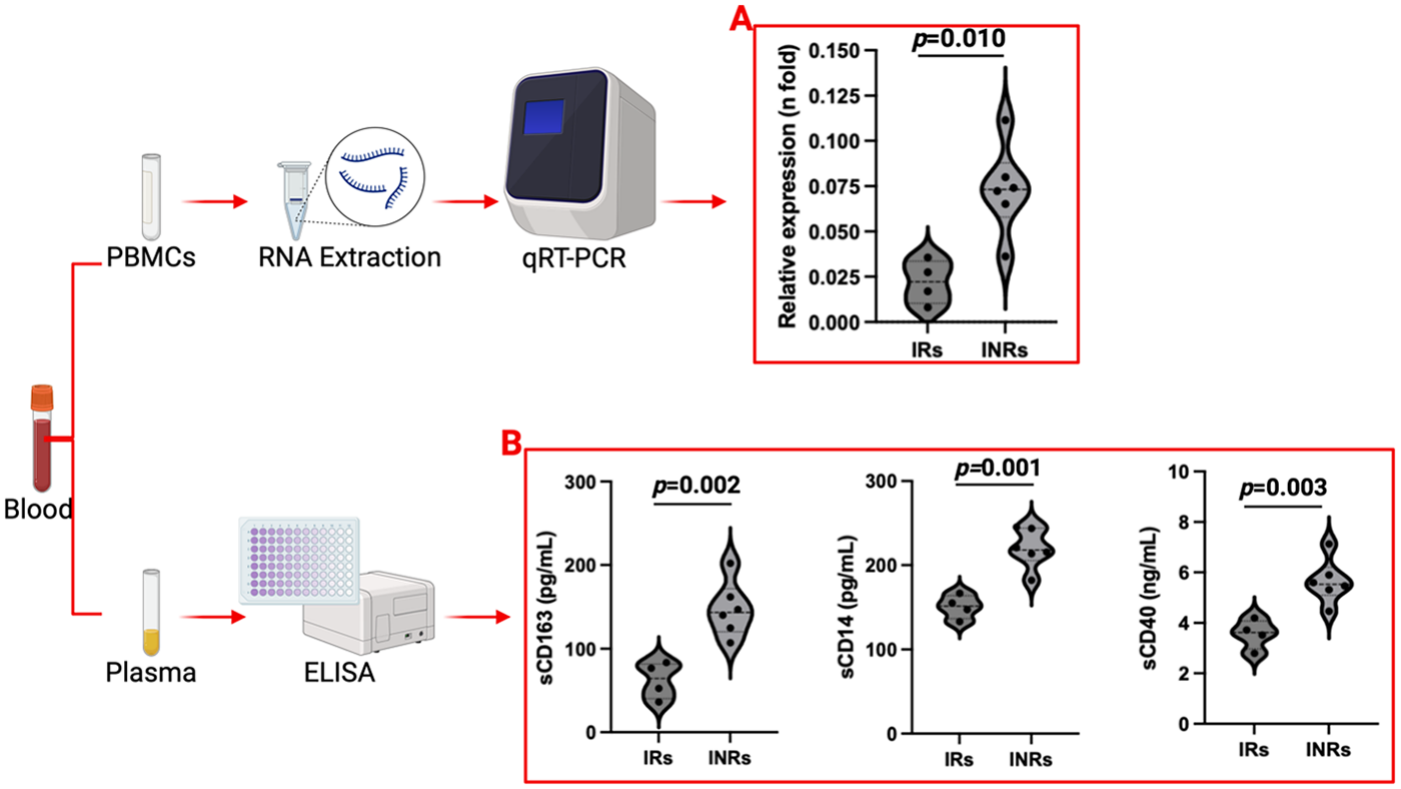
PSGL-1 expression **(A)** and plasma marker profiles **(B)** in ART-treated adults. INRs: Immunological non-responders; IRs: Immunological responders. Only plasma and PBMCs from 10 ART-treated individuals (4 IRs and 6 INRs) were considered.

In a preliminary investigation, our group has evaluated the expression of PSGL-1 in PBMCs (Figure 3A) and have studied the possible associations between the preceding markers and PSGL-1 expression in ART-treated PLWH. This investigative study used a few samples [selected randomly from IRs (>2 years ART; ≥200 CD4+ T-cells/µl; plasma HIV load <50 copies/ml) and INRs (>2 years ART; <200 CD4+ T-cells/µl; plasma HIV load <50 copies/ml); see Table 2] which were originally collected for a larger study investigating PSGL-1 expression in HIV positive individuals. For the general project, participants were excluded if they: (1) presented with an active opportunistic infection, HBV or HCV infection, or any coexisting chronic disease, (2) were found to have organ failure or who were found to be in a decompensated state, (3) were pregnant or breastfeeding, (4) were below 18 or above 60 years of age. Written informed consent were obtained from all study participants before blood sample collection. This study was reviewed and approved by the ethics committee of Chongqing Public Health Medical Center.

**Table 2:** Characteristics of the patients considered.

Characteristic	Treated HIV+	
	INRs	IRs
Sex		
Male [*n* (%)]	4 (66.7)	3 (75)
Female [*n* (%)]	2 (33.3)	1 (25)
Age [Median (IQR)]	51 (25)	55 (19)
HIV-1 viral load in copies/ml [Median (IQR)]	<50	<50
CD4+ T-cells/µl [Median (IQR)]	142.5 (88)	499 (50)
Platelet count ×10^9^/L [Median (IQR)]	185.5 (75)	182.5 (56)
Nadir CD4+ T-cells/µl [Median (IQR)]	97 (136)	434 (374)
ART period in years [Median (IQR)]	6.5 (7)	7 (5)

Data are median (IQR) or *n* (%). Treated HIV+, HIV positive patients receiving ART for more than 2 years; INRs, immunological non-responders; IRs, immunological responders.

#### 3.2.2 Blood sample management

For each participant, ten milliliters of blood was collected in EDTA tubes then transferred to the laboratory. There, CD4+ T-cell and CD8+ T-cell count were determined using flow cytometry (75), while platelet count was determined with a blood cell analyzer. Plasma was separated from whole blood using centrifugation and stored at −80°C. Finally, PBMCs were isolated by Ficoll density gradient centrifugation and stored at −80°C. Plasma and PBMCs used in this prospective analysis had been stored for at least six months before further analysis.

#### 3.2.3 Analysis of PSGL-1 expression and plasma marker concentration

PSGL-1 expression was determined using quantitative RT-PCR (qRT-PCR). Thus, total RNA from the stored PBMCs was isolated using the RNeasy Micro Kit (Qiagen, 74004, Germany), in accordance with the manufacturer's protocol. The NanoDrop 1,000 spectrophotometer was utilized to determine the concentration of extracted RNA, then 1 µg of RNA was used for cDNA synthesis using the Qiagen Quantitect Reverse Transcription kit (Qiagen, 205311, Germany). Quantitative RT-PCR was performed using the HotMaster Taq DNA polymerase formulation (Aidlab, China). Forward primers (TCCTCCTGTTGCTGATCCTACTG) and reverse primers (TACTCATATTCGGTGGCCTGTCT) were used to amplify PSGL-1, utilizing recognized methods that have been published in the contemporary literature (82). The housekeeping gene, GAPDH (forward: TCAAGGCTGAGAACGGGAAG; reverse: CGCCCCACTTGATTTTGGAG), was used as an internal control (86). To amplify each of these genes, the following protocol was used: 30 cycles, 30 s at 95°C, 30 s at 60°C, and 30 s at 72°C. Before and after these 30 cycles, we ran an initial denaturation of 3 min at 95°C and a final extension of 7 min at 72°C, respectively. Thermal cycling was executed on the LightCycler® 96 Thermal Cycler (Roche Diagnostics GmbH, Mannheim, Germany). Relative quantification was performed via the 2^−ΔΔCT^ method (87).

Enzyme-linked immunosorbent assay (ELISA) was used to determine the concentration of plasma markers. Briefly, using ELISA kits, the levels of soluble CD40 (sCD40) (Jiangsu Meibiao Biotechnology, MB-0039A), sCD14 (Jiangsu Meibiao Biotechnology, MB-3820A), and sCD163 (Jiangsu Meibiao Biotechnology, MB-3848A) in patient's plasma were determined. All experiments were conducted in strict accordance with each manufacturer's instructions. The SpectraMax ABS Plus microplate reader (monitored by SoftMax Pro7.1 software) was used to read absorbance and to quantify the expression of each marker.

#### 3.2.4 Data analysis

Continuous variables were expressed as mean (±standard deviation) if normally distributed, or as median [interquartile range (IQR)] if not. Categorical variables were presented as frequencies (percentages). Spearman's correlation test was used to determine the correlations between PSGL-1 expression and plasma markers of CVD and inflammation. The statistical significance level for all tests was defined as a *p*-value of <0.05.

#### 3.2.5 Preliminary results

We noted that sCD163, sCD14, and sCD40 levels are significantly higher in INRs compared to IRs (*p* < 0.05, Figure 3B). This implies that INRs are at higher risk of developing CVDs due to higher plasma levels of markers (inflammatory and CVD) and higher levels of PSGL-1, which upon further investigations may be found to be responsible for the increased formation of platelet-leukocyte and/or endothelial cell-leukocyte aggregates. Our findings are somewhat complementary to those observed by Dai et al. (76), except for the fact that our team considered PBMCs in general rather than CD4+ T-cells exclusively.

**Figure 4:**
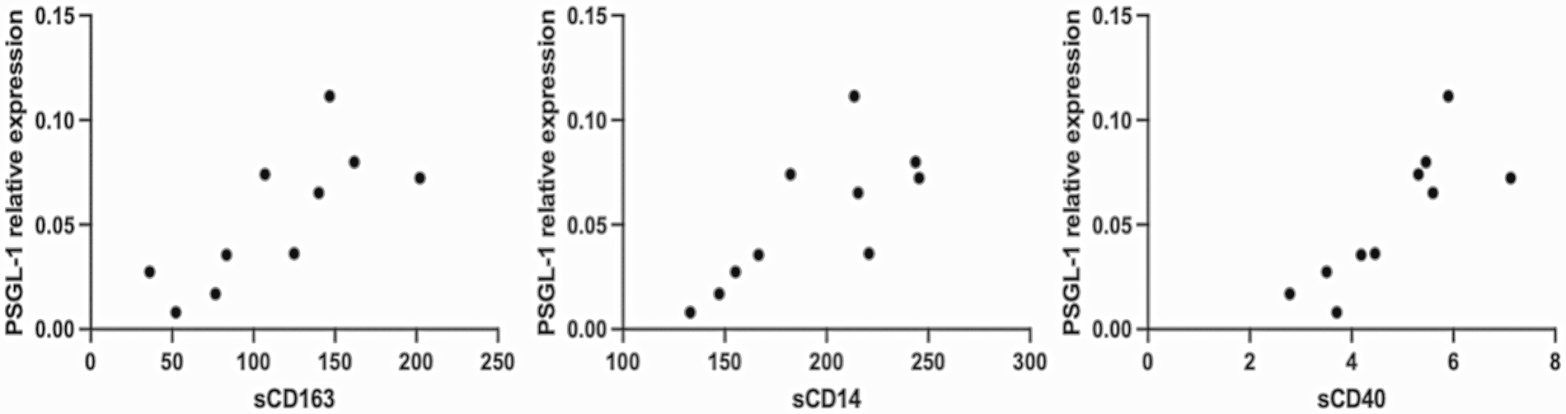
Representation of correlations. Only plasma and PBMCs from 10 ART-treated individuals (4 IRs and 6 INRs) were considered.

Interestingly, levels of sCD163, sCD14, and sCD40 show very strong positive correlations with PSGL-1 expression (Table 3 and Figure 4). Notably, PSGL-1, sCD163, sCD14, and sCD40 are already recognized as being associated with development of CVDs in the general HIV negative context. Thus, our preliminary results suggest that the levels of the preceding plasma markers and the expression of PSGL-1 may potentially indicate a higher risk for INRs to develop CVDs, when compared to IRs.

**Table 3:** Correlation of PSGL-1 with plasma markers of CVD and inflammation.

Category of patient	Variables	Correlation coefficient	*p-*value
Treated HIV+	PSGL-1-sCD163	0.81	0.005
	PSGL-1-sCD14	0.73	0.020
	PSGL-1-sCD40	0.83	0.005

Treated HIV+, ART-experienced patients stratified into INRs and IRs.

### 3.3 Interpretation

In past studies, researchers have observed that PSGL-1 functions as an HIV restriction factor (83). We have therefore postulated that the potential utilization of PSGL-1 as a target to treat HIV infection is a justifiably promising strategy (88, 89). However, recent evidence indicates that PSGL-1 expression is not fully beneficial in the preceding context, as PSGL-1 expression may also promote HIV transmission depending on the levels of PSGL-1 expression on immune cells (90). Indeed, it is known that when PSGL-1 is expressed at lower proportions on an HIV-infected cell, the produced virions may use the incorporated PSGL-1 in their membrane structure to infect HIV negative cells via PSGL-1/P-, E, or L-selectin attachment (90). Conversely, when PSGL-1 is overexpressed on HIV infected cells, a larger quantum of PSGL-1 is present on the virion membrane, which makes it challenging for new HIV virions to attach to and infect other healthy cells (90). Interestingly, this contemporary profile of PSGL-1 expression (based on the literature review delineated in the first part of this article) may imply an elevated risk of an increase in the pathogenesis of CVDs. Herein; we have observed that PBMCs from INRs display an overexpression of PSGL-1. This, if validated in future focused studies, may well explain why HIV-infected individuals who are INRs tend to develop a higher proportion of CVDs compared to IRs. Recently, our group has hypothesized that PSGL-1 may potentially be utilized as a biomarker for pathological conditions such as immunological status, inflammation/translocation, cell exhaustion, and cancers in HIV infection (91). In light of the preceding observations made in this article, we believe that it is likely that the spectrum of negative immunopathophysiological influence that PSGL-1 is capable of exerting may be much wider than is currently appreciated.

## 4 Conclusive remarks

This article presents evidence from the contemporary literature which shows that overexpression of PSGL-1 is associated with manifestations of CVD. Although there is a scarcity of published data on PSGL-1 expression in the HIV infection context, this article informs on a potentially neglected role of PSGL-1 in PLWH. Indeed, in juxtaposing previous evidence with clues observed in preliminary clinical investigations conducted by our team and others, it is quite valid to conceive that PSGL-1 may be considered as a possible marker for the monitoring of CVDs in ART-treated individuals in general. As has been observed, the elevated levels of PSGL-1 in INRs may expose these patients to a higher risk for the development of CVDs, compared to IRs. However, only future research with robust investigative approaches and large sample sizes will aid in validating this hypothesis. Apart from the relatively small sample sizes used in our prospective analysis, the present investigative approach has several other limitations, as in addition to determination of intracellular PSGL-1 expression, extracellular expression of PSGL-1 should technically also be determined during the investigative procedures. Indeed, for future investigations, analyses using flow cytometry will be more appropriate, as flow cytometry will depict true PSGL-1 expression, as opposed to mRNA quantification. In the present study, it was not possible to perform flow cytometry as the samples had already been stored at −80°C for at least 6 months. For accurate and valid flow cytometric results to be obtained in the context of this article, it is recommended that analysis of PSGL-1 expression be performed within 8 h after blood collection (92). Thus, due to the logistical constraints presented by our sampling, we chose to perform qRT-PCR on our samples, as the physical structure of PSGL-1 molecules on PBMCs were likely to be altered by the method and duration of sample storage, and thus would likely not be fully recognized by anti-PSGL-1 antibodies, and measured levels would therefore be underestimated via flow cytometric studies. Notably, this study has a further major limitation as only PSGL-1 expression was investigated. However, for leukocyte-platelet aggregate formation, in addition to PSGL-1, platelet activation is required. Thus, the expression of P-selectin on platelets and a study of potential correlations between P-selectin and PSGL-1 expression should be investigated in the future. Similarly, it is known that the levels of PSGL-1 expressed on leukocytes or CD4+ T-cells do not imply that all PSGL-1 will necessarily be engaged by P-selectin on platelets. Indeed, it is known that PSGL-1 requires glycosylation [formation of sialyl Lewis × (sLex)-capped O-glycans] before being able to bind to selectins, including P-selectin (93, 94). A profiling of the expression of the enzymes [fucosyltransferase-VII (FucT-VII) and fucosyltransferase-IV (FucT-IV), amongst others] responsible for that glycosylation would likely have furnished further critical evidence with respect to the mechanism associated with the formation of leukocyte-platelet aggregates in the context of HIV infection. Thus, investigations into the activities of such enzymes and a profiling of PSGL-1 glycosylation are warranted. Finally, investigations involving screening of PSGL-1 expression in ART-treated PLWH (IRs and INRs) and accurate stratification of PLWH with and without CVDs in these screened cohorts will be fundamental in the quest to reveal the precise role of PSGL-1 expression in those PLWH with CVDs, and indeed, the role that PSGL-1 expression plays in the development of CVDs in the general population.

If PSGL-1 is to be considered as a biomarker for CVDs in PLWH, it is essential to acknowledge that the feasibility, the cost, and additional challenges for PSGL-1 screening should be thoroughly analyzed. With respect to feasibility, we believe that PSGL-1 screening using flow cytometry is easily manageable. This could be done routinely as CD4+ T-cell counts are determined for each HIV positive patient. The cost of PSGL-1 antibodies is relatively accessible for laboratories (ranging from $100 to $600 for a tube of 100 tests). As such, the cost is unlikely to be prohibitive. However, the major challenge remains the confounding factors inherent to individual patients, among which there are BMI, age, potential coinfections, and the specific ART regimen being used, and others. For example, being overweight/obese with an elevated BMI is known to induce inflammation (95, 96), which may in turn influence PSGL-1 expression in ART-treated individuals. In the US, 22% and 5% of ART-treated individuals, respectively, become overweight or obese (97). PSGL-1 monitoring in such individuals may not be accurate at predicting the onset of CVDs. There are also questions around modern ART-regimens and their potential influence on PSGL-1 expression. It is known that some ART drugs induce an accumulation of adipose tissue and may therefore cause HIV positive individuals to become overweight/obese. For example, compared to NNRTI or PI-based regimens, INSTI-based regimens may lead to greater weight gain (98–102). Thus, it is likely that patients receiving INSTI-based regimens may have overexpression of PSGL-1. Patient age represents another confounding factor, as advanced age is also associated with an underlying proinflammatory state (103). Therefore, the effects of proinflammatory cytokines may be associated with overexpression of PSGL-1 on immune cells and may therefore distort results from the screening of PSGL-1 in older ART-treated individuals. PSGL-1 expression depending on gender and/or ethnicity is also worth studying. Potential differences may serve to help to distinguish the normal range from pathological levels in each group of individuals. Additionally, for HIV-infected individuals, comorbidities should be diligently assessed and reported, as they also may induce aberrant expression of PSGL-1. Nevertheless, in the light of information gleaned from the contemporary literature, we believe that establishing PSGL-1 as a potential biomarker for CVDs in PLWH is clinically feasible and practically informative, as it may aid in further assisting clinical practitioners to predict and monitor patients having a higher risk of developing CVDs.

## Data Availability

The datasets presented in this study can be found in online repositories. The names of the repository/repositories and accession number(s) can be found in the article/Supplementary Material.
