# Supplementary material for: P-selectin glycoprotein ligand-1 and cardiovascular diseases: from a general perspective to an HIV infection context
Source: Front Cardiovasc Med. 2025 Feb 18;12:1521158. doi: 10.3389/fcvm.2025.1521158 (PMC11876174; doi:10.3389/fcvm.2025.1521158)
Supplement: Supplementary file 1 [file Datasheet1.docx]

**Supplementary files**

**1. Context of study**

This prospective analysis explored the levels of PSGL-1 expressed in different categories of HIV-1-infected adults ≥18 years of age. It uses a few samples (selected randomly) which were collected for a larger study investigating PSGL-1 expression in HIV positive individuals. This study was reviewed and approved by the ethics committee of Chongqing Public Health Medical Center. Thus, HIV-positive immunological responders (IRs) and immunological non-responders (INRs) who were strictly compliant with prescribed ART over two continuous years were selected (plasma HIV load <50 copies/ml). IRs were defined as patients who received ART for at least 2 years and were able to surpass a specified CD4+ T-cell count threshold (≥200 CD4+ T-cells/µl). INRs were defined as patients having an extremely low baseline CD4+ T-cell count (CD4+ T-cells <200/µl) despite receiving ART for at least 2 years

For the general project, participants were excluded if they: (1) presented with an active opportunistic infection, HBV or HCV infection, or any coexisting chronic disease, (2) were found to have organ failure or who were found to be in a decompensated state, (3) were pregnant or breastfeeding, (4) were below 18 or above 60 years of age. Written informed consent were obtained from all study participants before blood sample collection.

**2. Samples collection and storage**

Ten milliliters (mL) of blood were collected from each participant in EDTA tubes, and was stored as 3 mL of plasma (stored at -80 ºC) and 1 mL of peripheral blood mononuclear cells (PBMCs, stored at -80 ºC). The samples were stored for at least six months before our prospective analysis.

Table 1. Characteristics of the patients considered

| Characteristic | Treated HIV+ | |
| --- | --- | --- |
|  | INRs | IRs |
| Sex  Male  Female | 4 (66.7)  2 (33.3) | 3 (75)  1 (25) |
| Age | 51 (25) | 55 (19) |
| HIV-1 viral load in copies/mL | <50 | <50 |
| CD4+ T-cells/µl | 142.5 (88) | 499 (50) |
| Platelet count x10^9^/L | 185.5(75) | 182.5(56) |
| Nadir CD4+ T-cells/µl | 97 (136) | 434 (374) |
| ART Period in years | 6.5 (7) | 7(5) |

Data are median (IQR) or n (%). New HIV+: ART-naïve newly diagnosed HIV-positive adults; Treated HIV+: HIV+ patients receiving ART for more than 2 years; INRs: Immunological non-responders; IRs: Immunological responders.

**3. PSGL-1 expression with quantitative RT-PCR (qRT-PCR)**

Total RNA from the stored PBMCs was isolated using the RNeasy Micro Kit (Qiagen, 74004, Germany), in accordance with the manufacturer’s protocol. The NanoDrop 1000 spectrophotometer was utilized to determine the concentration of extracted RNA, then 1µg of RNA was used for cDNA synthesis using the Qiagen Quantitect Reverse Transcription kit (Qiagen, 205311, Germany). Quantitative RT-PCR was performed using the HotMaster Taq DNA polymerase formulation (Aidlab, China). Forward primers (TCCTCCTGTTGCTGATCCTACTG) and reverse primers (TACTCATATTCGGTGGCCTGTCT) were used to amplify PSGL-1, utilizing recognized methods that have been published in contemporary literature ^17^. The housekeeping gene, GAPDH (forward: TCAAGGCTGAGAACGGGAAG; reverse: CGCCCCACTTGATTTTGGAG), was used as an internal control ^18^. To amplify each of these genes, the following protocol was used: 30 cycles, 30 sec at 95ºC, 30 sec at 60ºC, and 30 sec at 72ºC. Before and after these 30 cycles, we ran an initial denaturation of 3 min at 95ºC and a final extension of 7 min at 72ºC, respectively. Thermal cycling was executed on the LightCycler^®^ 96 Thermal Cycler (Roche Diagnostics GmbH, Mannheim, Germany). Relative quantification was performed via the 2^−ΔΔCT^ method ^19^.

**4. Plasma markers determined with enzyme-linked immunosorbent assay (ELISA)**

Using ELISA kits, the levels of soluble CD40 (sCD40) (Jiangsu Meibiao Biotechnology, MB-0039A), sCD14 (Jiangsu Meibiao Biotechnology, MB-3820A), and sCD163 (Jiangsu Meibiao Biotechnology, MB-3848A) in patient’s plasma were determined. All experiments were conducted in strict accordance with each manufacturer’s instructions. The SpectraMax ABS Plus microplate reader (monitored by SoftMax Pro7.1 software) was used to read absorbance and to quantify the expression of each marker.

**5. Statistical analysis**

Continuous variables are expressed as mean (±standard deviation) if normally distributed, or as median [interquartile range (IQR)] if not. Categorical variables are presented as frequencies (percentages). Spearman’s correlation test was used to determine the correlations between PSGL-1 expression and plasma markers of CVD and inflammation. The statistical significance level for all tests was defined as a *p*-value of <0.05.
